# Trust buffers price-related barriers to HPV vaccination among female college students: a cross-sectional study in China based on the 3C model

**DOI:** 10.3389/fpubh.2026.1848085

**Published:** 2026-07-14

**Authors:** Yiyi Zhuang, Jianye Zhao, Mei Wang, Qing Liu, Weiwei Sun, Yanping Sun, Kuimei Zhang, Xiaoxia Wang, Baojun Pan, Wenming Cao

**Affiliations:** 1 Health Industry Department, College of Health Industry, Xiamen Donghai Vocational and Technical College, Xiamen, Fujian, China; 2 Department of Gynecology, Pingshan District Central Hospital of Shenzhen, Shenzhen, Guangdong, China; 3 Changle People’s Hospital, Weifang, Shandong, China

**Keywords:** 3C model, female college students, HPV vaccine, trust, vaccination intention, vaccine hesitancy

## Abstract

**Purpose:**

HPV vaccine hesitancy is prevalent among female college students in China, yet how trust interacts with cost concerns remains unclear. This study, grounded in the 3C model, examined whether trust in healthcare professionals buffers price-related barriers and identified high-risk subgroups.

**Methods:**

A cross-sectional survey recruited 4,326 female college students from 12 Chinese provinces. Trust, price perception, and vaccination intention were measured. Interaction effects were tested using the Breslow-Day test; a decision tree model (CART) identified high-risk profiles.

**Results:**

Trust in healthcare professionals showed the strongest positive correlation with vaccination intention (*r*_s_ = 0.610, *p* < 0.001), with a clear dose–response pattern. A significant trust-by-price interaction emerged (*p* = 0.008): among low-trust respondents, high price was associated with substantially higher hesitancy (78.5% vs. 54.3%, *p* < 0.001); among high-trust respondents, the price effect was negligible (35.6% vs. 30.1%, *p* = 0.12). The most frequently cited reasons for hesitancy were high cost (53.5%), fear of side effects (52.7%), and limited vaccine knowledge (46.3%). Decision tree analysis identified “low trust and high price” as the dominant high-risk profile, accounting for 32.4% of all hesitant individuals.

**Conclusion:**

Trust can buffer price-related barriers to HPV vaccination. Stratified interventions should prioritize the “low trust and high price” group through combined trust-building and financial support.

## 1 Introduction

Cervical cancer (CC) ranks as the fourth most common malignancy among women worldwide, with an estimated 661,000 new cases and 348,000 deaths in 2022 (1). China bears roughly one-fifth of this global burden, and incidence rates are rising among younger women (2). Persistent infection with high-risk human papillomavirus (HPV) is the primary cause (3). As a key prevention strategy, the World Health Organization (WHO) has set a 2030 target: 90% of girls fully vaccinated against HPV by age 15 (4). Yet despite proven vaccine safety and efficacy, “vaccine hesitancy,” defined as delay or refusal despite availability, was identified as a major challenge to immunization programs, prompting the WHO to prioritize vaccine confidence in its Immunization Agenda 2031 (5).

Female college students (FCSs) may face elevated HPV risk, as previous studies have reported that this life stage is often associated with increased opportunities for HPV exposure, including the initiation or change of sexual partnerships, though individual experiences vary substantially (6). In China, however, their HPV vaccination rate remains below 20%, with hesitancy rates estimated between 30 and 50% (7). Most prior research has relied on the Health Belief Model (HBM), emphasizing individual perceptions of susceptibility, severity, benefits, and barriers (8). While valuable, the HBM pays limited attention to broader structural factors such as trust in the healthcare system or vaccine accessibility.

The WHO-convened “3C model” offers a more integrated framework, identifying three core drivers of hesitancy: Complacency (low perceived disease risk), Confidence (trust in vaccine safety, efficacy, and the system delivering it), and Convenience (accessibility and affordability) (9). Compared to the HBM, the 3C model provides theoretical advantages: the HBM’s “perceived barriers” construct conflates cost concerns, access difficulties, and trust-related safety doubts into a single dimension, whereas the 3C model isolates “Confidence” (trust in vaccine safety, efficacy, and the healthcare system) as a distinct driver. This separation is important because trust operates through relational and informational channels that differ fundamentally from material barriers like price or distance. The 3C model explicitly incorporates “Convenience” (affordability and accessibility) alongside “Confidence” “making it better suited to settings where resource constraints and trust deficits co-exist and may interact. Although the 3C model has been applied in European and North American HPV studies, its validity among FCSs, particularly regarding the interplay between trust and price, remains underexplored (10).

Trust has gained increasing attention in vaccination research. A large-scale study drawing on China’s General Social Survey found that trust, particularly in medical professionals and government, predicted lower vaccine hesitancy more strongly than generalized or online trust (11). Systematic reviews of FCSs echo this: concerns about vaccine safety and side effects (both trust-related) consistently emerge as dominant influences on willingness (12). For HPV specifically, beliefs about vaccine efficacy and safety strongly predict uptake, alongside information gaps and family influences (13). Even under fully subsidized programs, Chinese parents’ trust in domestic vaccines significantly affects their willingness to accept HPV vaccination for daughters, suggesting trust operates independently of cost (14).

Our team’s earlier work identified rural–urban registration, only-child status, and academic major as correlates of HPV vaccination among FCSs (15). A Shenzhen-based study of reproductive-age women found that 24.6% expressed hesitancy, with price perception and safety concerns as key drivers (16). Healthcare professionals’ recommendations carry particular weight as authoritative information sources, yet the dose–response nature of trust and its moderating role under varying price perceptions remain unclear.

Guided by the 3C framework, this large-sample survey aims to describe current HPV vaccination intention and hesitancy patterns among FCSs; examine whether trust, especially in healthcare professionals, functions as a core driver, and test for dose–response relationships; assess whether trust buffers the negative impact of price; and identify high-risk subgroups via decision tree modeling to inform precise, stratified interventions.

## 2 Materials and methods

### 2.1 Study design and participants

This cross-sectional survey was conducted from January 2025 to January 2026. A total of 4,326 FCSs were recruited from 24 universities across 12 Chinese provinces (Guangdong, Fujian, Zhejiang, Jiangsu, Shandong, Beijing, Shanghai, Hubei, Sichuan, Shaanxi, Henan, and Liaoning) using convenience sampling. Participating institutions included 9 comprehensive universities, 8 medical universities/colleges, and 7 vocational/technical colleges. Recruitment was conducted through university health centers, student affairs offices, and course-based announcements; online recruitment via university WeChat public accounts supplemented in-person recruitment. Eligible participants were full-time female students enrolled in associate, undergraduate, or postgraduate programs who provided informed consent. We excluded those with incomplete questionnaires (>10% missing items) or inconsistent logical responses. Of 4,550 distributed questionnaires, 4,326 were valid (response rate: 95.1%). Sample size was determined using Kendall’s method: for 20 main analytic variables, we required 10–20 times the variable count, plus 10% allowance for invalid responses, yielding a minimum of 440. Our achieved sample substantially exceeded this.

### 2.2 Survey instruments

This study developed a structured questionnaire based on literature review and expert consultation, pretested for reliability and validity. The questionnaire was administered in simplified Chinese, the native language of all participants. The development process included: (1) Item generation: an initial pool of 45 items derived from PubMed/CNKI/Google Scholar literature (2020–2024) and WHO 3C model operationalization guides; (2) Expert review: five experts (two public health professors, two gynecologists, one health psychologist) assessed content validity, yielding item-level Content Validity Index (I-CVI) ranging from 0.80 to 1.00 and scale-level CVI (S-CVI/Ave) of 0.92; (3) Pilot testing (n = 30) confirmed readability, test–retest reliability (ICC = 0.78–0.85 over 2-week interval), and internal consistency (Cronbach’s *α* = 0.81–0.89). A detailed mapping of questionnaire items to the 3C dimensions (Complacency, Confidence, Convenience) is available from the corresponding author upon request. It included:

#### 2.2.1 Demographic characteristics

Age, educational level (associate degree/undergraduate/postgraduates), household registration (rural/urban), only-child status (yes/no), major (medical/non-medical), and primary income source (parental support/part-time work/family or friend support/student loans/scholarships/other).

#### 2.2.2 HPV vaccination history and intention

Prior vaccination (yes/no); vaccination intention rated on a 5-point Likert scale (1 = very unwilling to 5 = very willing); self-payment hesitancy assessed by asking: “Would you hesitate to receive the HPV vaccine if it required self-payment?” (yes/no).

#### 2.2.3 Trust perceptions

Four items rated on 5-point Likert scales (1 = very distrustful to 5 = very trusting): (1) Trust in healthcare professionals’ vaccination recommendations; (2) Trust in domestic bivalent HPV vaccine safety; (3) Trust in imported HPV vaccine safety; (4) Trust in vaccine effectiveness for cervical cancer prevention. Exploratory factor analysis extracted one factor explaining 72.4% of variance (loadings: 0.78–0.89). Cronbach’s *α* was 0.87, indicating strong internal consistency.

#### 2.2.4 Price perception

Perceptions of imported 9-valent vaccine price (too low/appropriate/too high); perceptions of domestic 2-valent vaccine price (too low/appropriate/too high); acceptable price range. At the time of the study (January 2025–January 2026), the actual market prices in China were: domestic bivalent HPV vaccine ¥360 per dose (approx. $53); imported 9-valent HPV vaccine ¥1,330 per dose (approx. $195).

#### 2.2.5 Reasons for hesitancy (multiple choice)

Ten options including high cost, fear of side effects, insufficient vaccine knowledge, etc.

### 2.3 Quality control

Investigators received uniform training before fieldwork. Questionnaires were self-administered and anonymous, with on-site verification. Data were double-entered and logic-checked. A pilot test (*n* = 30) confirmed readability and internal consistency (Cronbach’s *α*: 0.81–0.89).

### 2.4 Statistical analysis

Statistical analyses were performed using SPSS 26.0 and R 4.2.1. Descriptive statistics were computed, with continuous variables presented as mean ± standard deviation or median (interquartile range) and categorical variables as frequencies and percentages. For univariate analyses, chi-square tests (with Cramer’s *V* effect sizes) were used to compare vaccination intention across demographic and socioeconomic groups, while Spearman’s rank correlation assessed associations between trust and intention. Differences in trust levels across subgroups were examined using ANOVA, *t*-tests, or nonparametric alternatives as appropriate. Variables achieving statistical significance (*p* < 0.05) in univariate analyses were subsequently entered into stepwise multiple linear regression (with vaccination intention as the outcome) and stepwise binary logistic regression (for self-payment hesitancy). Regression coefficients (*B*), standardized coefficients (*β*), odds ratios (OR), 95% confidence intervals (CI), and variance inflation factors (VIF) were reported, with the Hosmer–Lemeshow test and Nagelkerke *R*^2^ used to evaluate logistic model fit. Interaction effects between trust in healthcare professionals and price on self-payment hesitancy were examined using the Breslow–Day test, followed by stratified chi-square analyses. A decision tree model based on the CART algorithm was also constructed to predict self-payment hesitancy, incorporating all significant univariate predictors; this approach was chosen for its capacity to detect nonlinear relationships and higher-order interactions without imposing prior distributional assumptions, yielding visually interpretable classification rules. The tree was grown with a maximum depth of five, a minimum parent node size of 100, and a minimum child node size of 50, and was subsequently pruned using 10-fold cross-validation to mitigate overfitting. Mediation analysis was conducted using bias-corrected bootstrap resampling (5,000 samples) to test whether trust in the domestic bivalent vaccine mediated the relationship between income source and hesitancy, a method that does not assume normality and provides robust confidence interval estimates. Finally, multiple response analysis summarized the frequencies and case percentages of reasons cited for hesitancy. All statistical tests were two-tailed with a significance threshold of *α* = 0.05.

### 2.5 Handling of trust categorization across analyses

To ensure transparency and reproducibility, trust in healthcare professionals (original 5-point Likert: 1 = very distrustful to 5 = very trusting) was treated differently depending on the analysis: (1) In the interaction analysis (Breslow-Day test), neutral responses (score = 3, *n* = 847, 19.6%) were excluded to create a clean contrast between low-trust (scores 1–2) and high-trust (scores 4–5) groups. (2) In the decision tree (CART) analysis, neutral responses were retained as a separate category because the algorithm can naturally handle three-level ordinal variables, and preserving the neutral group allowed detection of distinct risk patterns. (3) In regression analyses (linear and logistic), the original 5-point scale was treated as a continuous variable to preserve full variance.

### 2.6 Ethical considerations

The study was approved by the Ethics Committee of Shenzhen Pingshan District Central Hospital (PSZXYY-2024-334). All participants provided written informed consent. Anonymity was guaranteed, and data were used solely for research.

## 3 Results

### 3.1 Sample characteristics

**Figure 1:**
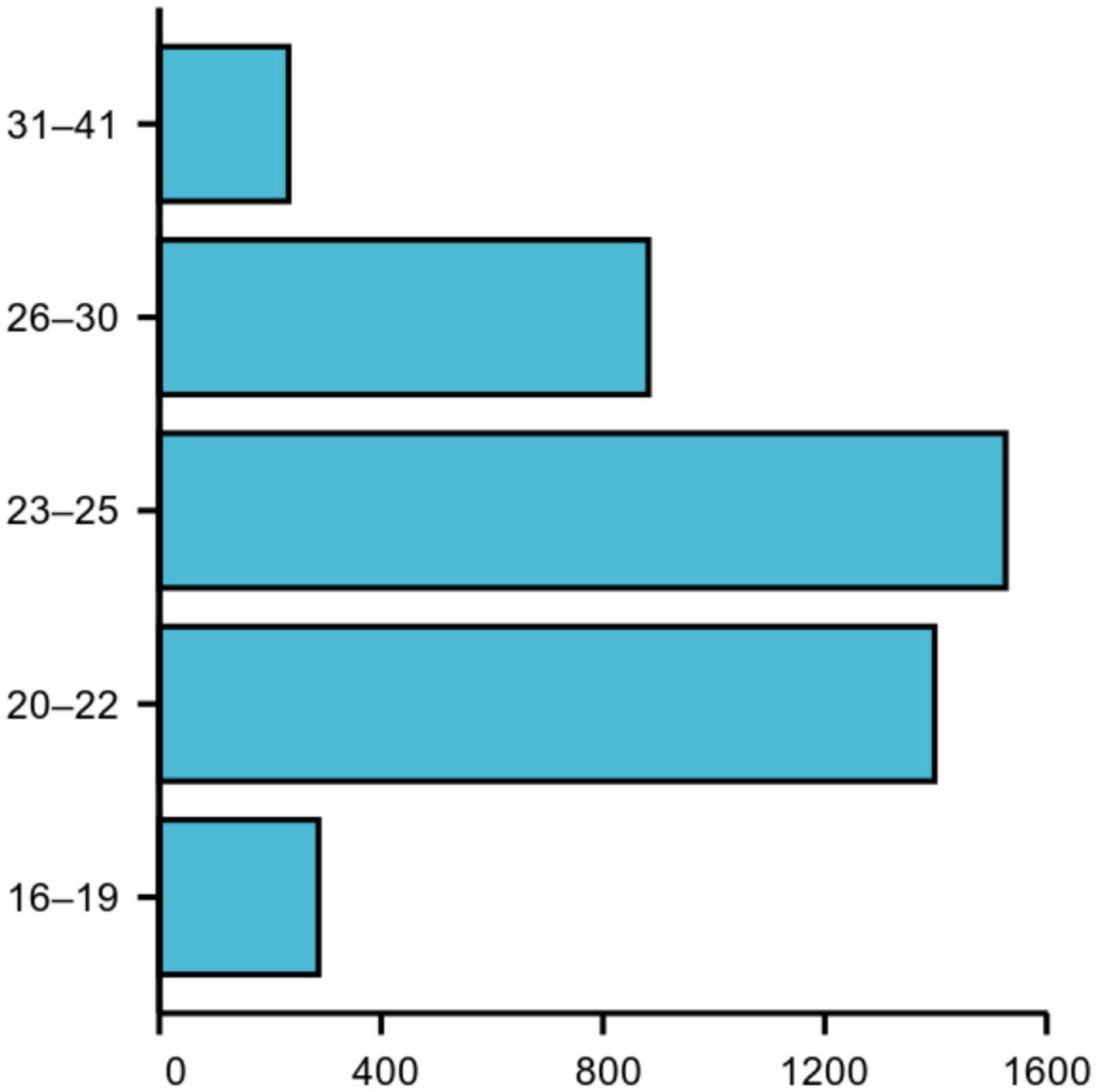
Age-stratified trends in high vaccination intention and self-payment hesitancy. High intention (very/somewhat willing) by age group with 95% confidence intervals (error bars). High intention was highest among participants aged 20–22 years (60%) and lowest among those aged ≥26 years (32%), showing a significant decline after age 25 (*χ*^2^ trend = 245.6, *p* < 0.001). Self-payment hesitancy by age group with 95% confidence intervals. Hesitancy was lower in the 16–25 age range (24–28%) but increased sharply to 52–56% in participants aged ≥26 years. Data are derived from Table 2 (*N* = 4,326). The *x*-axis represents age groups in years.

The 4,326 participants had a mean age of 24.64 ± 3.28 years (median: 25.0; range: 16–41). Overall, 76.75% had received HPV vaccination. Vaccination intention was distributed as follows: 47.3% expressed high intention (very/somewhat willing), 32.6% reported low intention (very/somewhat unwilling), and 20.1% were uncertain. Self-payment hesitancy was reported by 34.5%. Table 1 presents full demographic details. The sample showed balanced representation across educational levels, household registration, only-child status, major, and income sources. However, due to the convenience sampling design, these characteristics should not be overinterpreted as evidence of population-level generalizability; rather, they reflect the diversity within the recruited sample. Detailed age-stratified analyses are presented in Section 3.7 (Table 2 and Figure 1), and a comparison between vaccinated and unvaccinated participants is provided in Supplementary Table S1.

**Table 1:** Sample characteristics (*N* = 4,326).

Variable	Category	*N*	%
Age (years), mean ± SD			24.64 ± 3.28
Grade	Associate degree	1,182	27.3
	Undergraduate	2004	46.3
	Master’s or higher (postgraduates)	1,140	26.4
Household registration	Rural	2,280	52.7
	Urban	2046	47.3
Only child	Yes	2,597	60.0
	No	1729	40.0
Major	Medical	1856	42.9
	Non-medical	2,470	57.1
Income source	Parental support	2,696	62.3
	Part-time income	737	17.0
	Family/friend support	304	7.0
	Student loan	289	6.7
	Scholarship	199	4.6
	Other	101	2.3
Received HPV vaccine	Yes	3,320	76.8
	No	1,006	23.2
Vaccination intention	Very unwilling	430	9.9
	Somewhat unwilling	982	22.7
	Uncertain	869	20.1
	Somewhat willing	904	20.9
	Very willing	1,141	26.4
Self-payment hesitancy	Hesitant	1,494	34.5
	Not hesitant	2,832	65.5

Age range: 16–41 years; percentages are valid percentages.

**Table 2:** Age distribution and age-stratified vaccination intention and hesitancy (*N* = 4,326).

Age group (years)	*N* (%)	Very unwilling *N* (%)	Somewhat unwilling *N* (%)	Uncertain *N* (%)	Somewhat willing *N* (%)	Very willing *N* (%)	High intention *N* (%)	Self-payment hesitancy *N* (%)
16–19	287 (6.6)	34 (11.8)	52 (18.1)	40 (13.9)	57 (19.9)	104 (36.2)	161 (56.1)	80 (27.9)
20–22	1,398 (32.3)	126 (9.0)	210 (15.0)	224 (16.0)	308 (22.0)	530 (37.9)	838 (59.9)	363 (26.0)
23–25	1,526 (35.3)	122 (8.0)	183 (12.0)	366 (24.0)	320 (21.0)	535 (35.1)	855 (56.0)	366 (24.0)
26–30	882 (20.4)	159 (18.0)	247 (28.0)	194 (22.0)	124 (14.1)	158 (17.9)	282 (32.0)	459 (52.0)
31–41	233 (5.4)	65 (27.9)	70 (30.0)	23 (9.9)	42 (18.0)	33 (14.2)	75 (32.2)	130 (55.8)
Total	4,326 (100)	430 (9.9)	982 (22.7)	869 (20.1)	904 (20.9)	1,141 (26.4)	2045 (47.3)	1,494 (34.5)

All values are presented as frequency (row percentage). Row percentages were calculated with the total number of participants in each age group as the denominator. High intention = very/somewhat willing.

### 3.2 Univariate analyses

Chi-square tests revealed 14 factors significantly associated with vaccination intention (5-point ordinal). Trust-related variables yielded the largest chi-square values (peak: 1979.04) and Cramer’s *V* = 0.36 (medium-to-large effect), confirming trust as a core driver. Demographic factors (grade: *χ*^2^ = 149.64, *p* < 0.001, *V* = 0.13) and price perceptions followed. Nonsignificant factors included household registration (*p* = 0.075) and perceived need for school-based courses (*p* = 0.382). Spearman correlations showed that trust in healthcare professionals had the strongest association with intention (*r*_s_ = 0.610, *p* < 0.001), followed by trust in imported vaccines (0.592) and domestic bivalent vaccines (0.589). Price acceptability showed a weak negative correlation (*r*_s_ = −0.034, *p* = 0.027). Trust levels varied demographically: higher among associate/undergraduate students than postgraduates (*p* < 0.001); higher among those with parental financial support (*p* < 0.001); paradoxically, higher among those expressing hesitancy (*p* < 0.001). Among all variables, trust-related factors showed the strongest associations with vaccination intention, supporting their centrality in the 3C model.

### 3.3 Dose–response relationship: trust and intention

**Figure 2:**
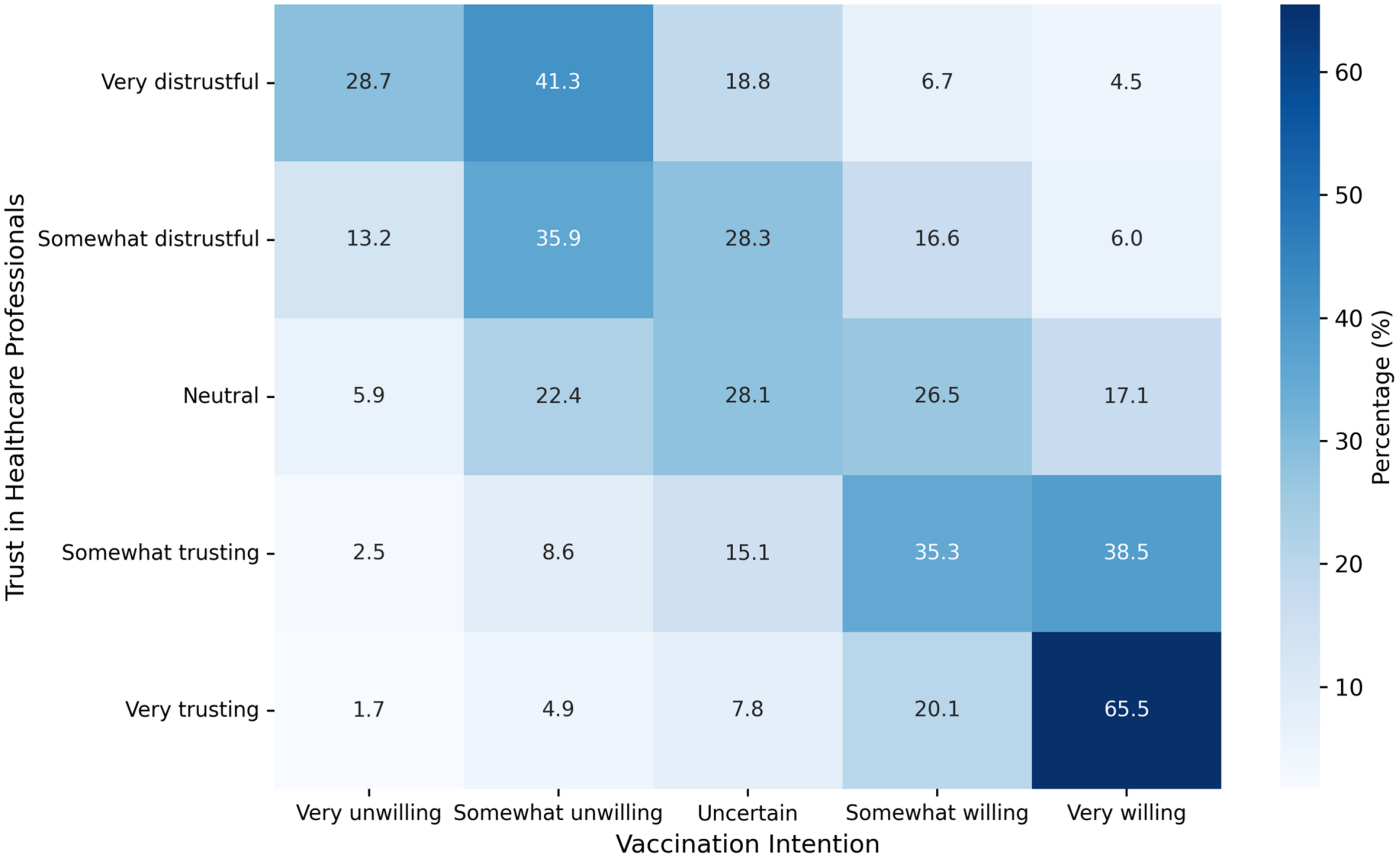
Trust in healthcare professionals and vaccination intention. *χ*^2^ = 1979.04, df = 16, *p* < 0.001; row percentages shown.

To examine the dose–response pattern, this study cross-tabulated healthcare professional trust with vaccination intention (Figure 2). As trust increased from “very distrustful” to “very trusting,” the proportion “very willing” rose monotonically from 4.5 to 65.5%. This graded association reinforces trust’s centrality. The monotonic increase in willingness across trust levels supports a dose–response relationship (Table 3).

**Table 3:** Significant univariate associations with vaccination intention.

Domain	Variable	*χ* ^2^	df	*p*	Cramer’s *V*
Demographics	Grade	149.64	8	<0.001	0.13
	Only child	11.66	4	0.020	0.05
	Major background	9.99	4	0.041	0.05
	Income source	115.80	40	<0.001	0.11
Trust factors	Trust in domestic bivalent vaccine	1752.21	16	<0.001	0.35
	Trust in imported HPV vaccine	1824.16	16	<0.001	0.36
	Trust in healthcare professionals	1979.04	16	<0.001	0.36
	Trust in preventive efficacy	1582.95	16	<0.001	0.34
Price/behavior	9-valent price perception	68.42	8	<0.001	0.10
	2-valent price perception	34.42	8	<0.001	0.08
	Acceptable price	66.35	20	<0.001	0.09
	Self-payment hesitancy	51.68	4	<0.001	0.08

Cramer’s *V* effect size (0.1 small, 0.3 moderate, 0.5 large). Non-significant factors included household registration (*p* = 0.075) and the necessity of school curriculum (*p* = 0.382).

### 3.4 Multiple linear regression: vaccination intention

With vaccination intention as outcome, this study entered demographic, trust, price, and hesitancy variables into stepwise linear regression (Table 4). The model explained a substantial proportion of variance (*R*^2^ = 0.488; adjusted *R*^2^ = 0.487; *F* = 456.289; *p* < 0.001). All VIFs < 2, indicating no multicollinearity. Trust perceptions remained the strongest predictors after adjustment: healthcare professional trust (*B* = 0.273, *β* = 0.284, *p* < 0.001), imported vaccine trust (*B* = 0.250, *β* = 0.247, *p* < 0.001), and domestic bivalent trust (*B* = 0.247, *β* = 0.257, *p* < 0.001) all positively predicted intention. Among demographics, older age (*B* = −0.021), postgraduate status (*B* = −0.116), and medical major (*B* = –0.082, *β* = −0.030, *p* = 0.006) were associated with lower intention (all *p* < 0.01). Income source, price acceptability, and hesitancy status became nonsignificant after controlling for trust.

**Table 4:** Multiple linear regression: vaccination intention.

Variable	*B*	SE	*β*	*t*	*p*	VIF
Constant	1.568	0.142	—	11.052	<0.001	—
Healthcare trust	0.273	0.014	0.284	18.954	<0.001	1.886
Imported vaccine trust	0.250	0.015	0.247	16.796	<0.001	1.816
Domestic bivalent trust	0.247	0.014	0.257	17.414	<0.001	1.828
Age	−0.021	0.005	−0.052	−4.355	<0.001	1.201
Postgraduate (vs. lower)	−0.116	0.035	−0.038	−3.352	0.001	1.088
Medical major (vs. non-medical)	−0.082	0.030	−0.030	−2.754	0.006	1.020
Non-parental income	−0.048	0.031	−0.017	−1.548	0.122	1.074
Price acceptability	0.007	0.011	0.006	0.594	0.553	1.007
Hesitancy (yes)	0.009	0.031	0.003	0.295	0.768	1.042

*R*^2^ = 0.488, adjusted R^2^ = 0.487, F = 456.289, p < 0.001. Variable coding: Grade (postgraduate = 1, associate degree or undergraduate = 0), major (medical = 1, non-medical = 0), Economic status (other = 1, parental support = 0), hesitancy (yes = 1, no = 0).

### 3.5 Binary logistic regression: self-payment hesitancy

With self-payment hesitancy as outcome, stepwise logistic regression (Table 5) yielded a significant model (Nagelkerke *R*^2^ = 0.048). Age emerged as the strongest negative predictor (OR = 0.903; 95% CI: 0.885–0.922; *p* < 0.001): each additional year reduced hesitancy odds by 9.7%. Notably, higher trust in imported vaccines slightly increased hesitancy odds (OR = 1.080; 95% CI: 1.028–1.135; *p* = 0.002), though the effect size was small. Students with non-parental income sources had lower hesitancy odds (OR = 0.842; *p* = 0.015).

**Table 5:** Logistic regression: self-payment hesitancy.

Variable	*B*	SE	Wald	*p*	OR	95% CI
Age	−0.102	0.010	95.17	<0.001	0.903	0.885–0.922
Imported vaccine trust	0.077	0.025	9.18	0.002	1.080	1.028–1.135
Non-parental income	−0.172	0.071	5.93	0.015	0.842	0.733–0.967
Constant	1.426	0.259	30.28	<0.001	4.161	—

–2LL = 5032.45; Nagelkerke *R*^2^ = 0.048; model *χ*^2^ = 153.62, *p* < 0.001.

### 3.6 Reasons for hesitancy

Multiple response analysis (Table 6) showed that the most common reasons were “vaccine price too high” (53.5%), “fear of side effects” (52.7%), and “lack of vaccine knowledge” (46.3%). Other notable barriers included inconvenient appointment procedures (44.3%), perceived low cervical cancer risk (40.3%), and limited vaccine availability (40.8%). The total case percentage (402.5%) indicated an average of 4.0 reasons per hesitant individual.

**Table 6:** Reasons for HPV vaccine hesitancy (multiple responses).

Reason	Responses (*N*)	Case %
Vaccine price too high	2,314	53.5
Fear of side effects	2,280	52.7
Lack of vaccine knowledge	2004	46.3
Inconvenient appointment procedures	1917	44.3
Vaccine not widely available	1763	40.8
Believe cervical cancer risk is low	1744	40.3
Doubt vaccine’s cancer prevention ability	1,661	38.4
Lack of family/friend support	1,468	33.9
Fear of injection pain	1,373	31.7
Other	889	20.6
Total	17,413	402.5*

*Total exceeds 100% due to multiple responses; average 4.0 reasons per person.

### 3.7 Age distribution and age-stratified analysis

Table 2 presents the detailed age distribution of the sample, along with age-stratified vaccination intention and self-payment hesitancy. In Table 2, all percentages are row percentages, calculated using the total number of participants in each age group as the denominator. The majority of participants (74.2%) were aged 20–25 years, representing the primary catch-up vaccination age range in China. High intention (very/somewhat willing) was highest among participants aged 20–22 years (60%) and lowest among those aged ≥26 years (32%), showing a clear decline after age 25 (*χ*^2^ trend = 245.6, *p* < 0.001). Self-payment hesitancy showed a U-shaped pattern: low in younger groups (28% at 16–19, 26% at 20–22, 24% at 23–25) but sharply higher in older groups (52% at 26–30, 56% at 31–41). Uncertainty (“uncertain” response) peaked in the 23–25 age group (24%), suggesting that students at the transition from undergraduate to postgraduate or workforce experience the most decisional conflict (Figure 1).

### 3.8 Comparison between vaccinated and unvaccinated participants

Vaccinated participants (76.8% of the sample) reported significantly higher trust across all four trust measures (Cohen’s *d* = 0.45–0.58), much lower self-payment hesitancy (18.3% vs. 88.1%, Cramer’s *V* = 0.48), and higher vaccination intention (Cohen’s *d* = 1.06) compared to unvaccinated participants. Medical students were overrepresented in the vaccinated group (44.2% vs. 38.6%, *p* = 0.002). Full details are provided in Supplementary Table S1.

### 3.9 Trust and price interaction

**Figure 3:**
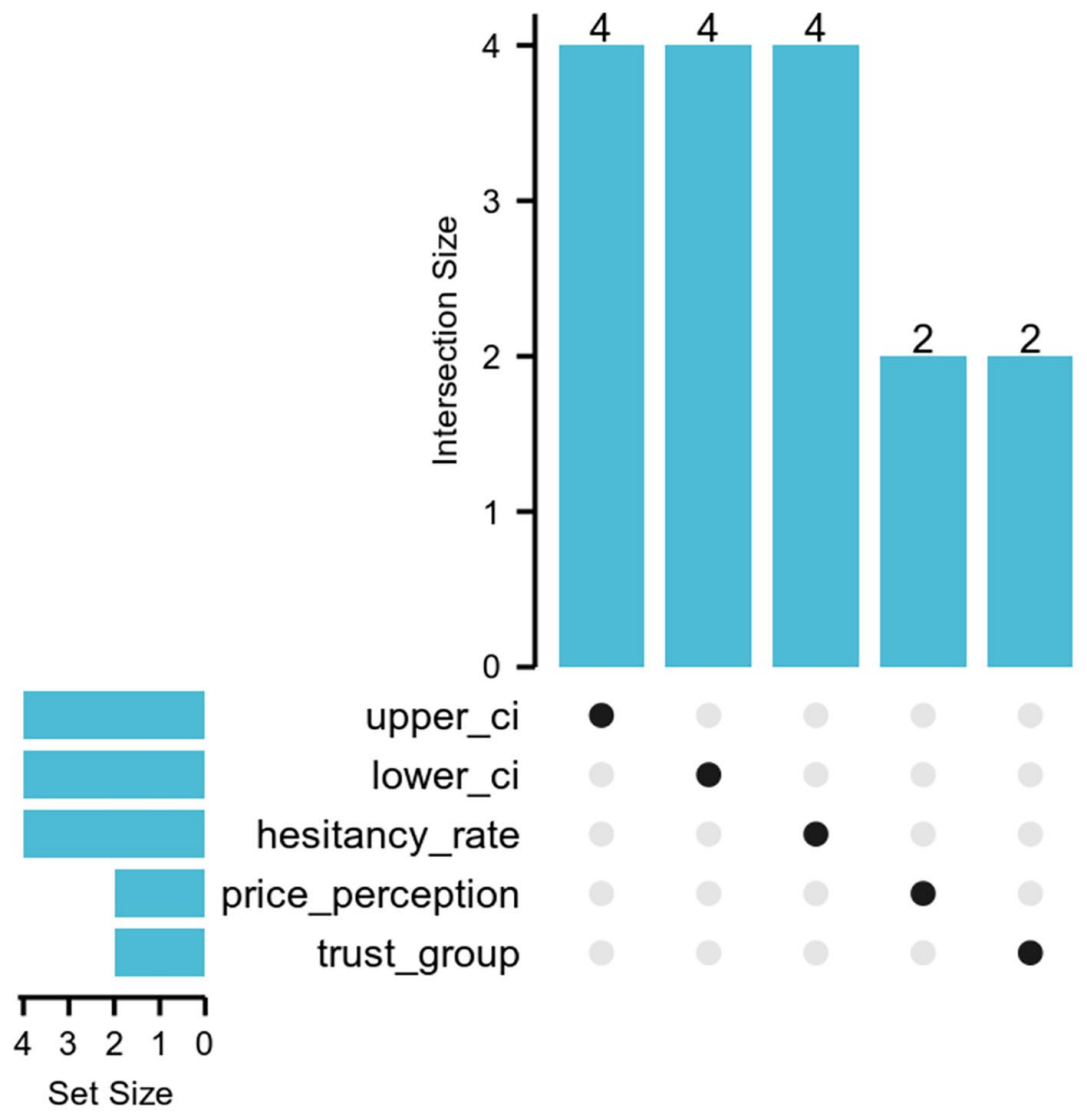
Interaction effect between trust in healthcare professionals and price perception on HPV vaccine hesitancy. Breslow-Day test for interaction: *χ*^2^ = 7.12, *p* = 0.008. Low trust group includes respondents who reported being “very distrustful” or “somewhat distrustful”; high trust group includes “somewhat trusting” or “very trusting.” High price perception refers to those who rated the vaccine price as “too high”; moderate/low price includes “appropriate” or “too low.” Percentages represent the proportion of hesitant individuals within each subgroup.

We tested for interaction between trust in healthcare professionals and price perception using the Breslow-Day test (Figure 3). Among participants who rated the vaccine price as “too high,” 71.3% relied on parental support, 15.8% on part-time income, and only 5.1% on scholarships, indicating alignment between price perception and financial status (data not tabulated). The Breslow–Day test confirmed significant effect modification (*p* = 0.008). In the low-trust group, high price dramatically increased hesitancy (78.5% vs. 54.3%, *p* < 0.001). In the high-trust group, the price effect was negligible (35.6% vs. 30.1%, *p* = 0.12). This pattern reveals trust’s buffering role against price barriers.

### 3.10 Decision tree: high-risk group identification

**Figure 4:**
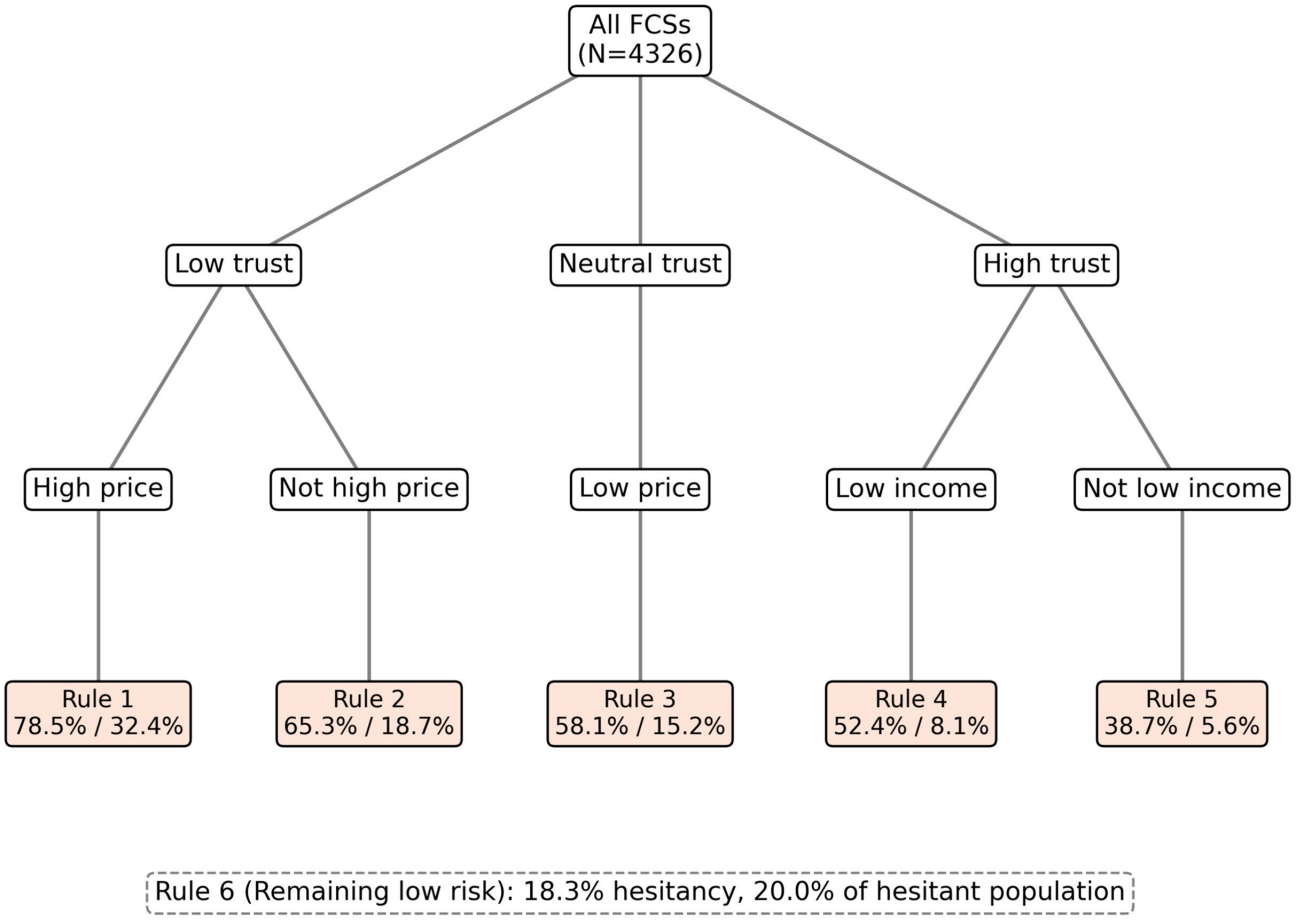
Classification rules from decision tree analysis identifying high-risk subgroups for HPV vaccine hesitancy. Training set *n* = 3,026 (70%), validation set *n* = 1,300 (30%), 10-fold cross-validation. CART algorithm; max depth = 5; min parent node = 100; min child node = 50; 10-fold cross-validation. The overall prediction accuracy was 78.3%, with an area under the curve (AUC) of 0.81.

The CART decision tree model (self-payment hesitancy as outcome) achieved 78.3% overall prediction accuracy and an area under the ROC curve of 0.81, indicating good discriminative performance. The model generated five high-risk rules covering 68.7% of hesitant individuals (Figure 4). Rule 1 (Distrust + High price) emerged as the core high-risk profile, with 78.5% hesitancy, accounting for 32.4% of all hesitant participants. Rules 2–5 further stratified risk by grade, acceptable price, major, income source, and age. Refer to Table 7.

**Table 7:** High-risk subgroups identified by CART decision tree analysis for HPV vaccine hesitancy.

Rule	Subgroup definition	*N*	Hesitancy rate (%)	All hesitant individuals (%)
Rule 1	Low trust + High price	815	78.5	32.4
Rule 2	Low trust + Not high price + Junior grade	312	62.3	12.1
Rule 3	Neutral trust + Low price + Non-medical major	428	54.1	14.3
Rule 4	High trust + Low income (non-parental support)	267	51.9	8.7
Rule 5	High trust + Parental support + Age < 22 years	356	48.6	10.6
Others	All remaining combinations	2,148	18.4	21.9
Total		4,326	34.5	100.0

Low trust = scores 1–2; neutral trust = score 3; high trust = scores 4–5. High price = perceived vaccine price as “too high”; junior grade = associate degree or undergraduate years 1–2.

### 3.11 Mediation analysis

Bootstrap mediation analysis (5,000 resamples) showed that trust in domestic bivalent vaccine partially mediated the relationship between income source and hesitancy (indirect effect = −0.009; 95% CI: −0.013 to −0.005). The path diagram is provided in Supplementary Figure S1.

### 3.12 Paradoxical finding: higher trust among hesitant individuals

Contrary to expectation, participants who reported self-payment hesitancy (n = 1,494, 34.5%) showed slightly but significantly higher trust in healthcare professionals than non-hesitant participants (mean 3.98 vs. 3.72, Cohen’s *d* = 0.30, *p* < 0.001). This pattern was consistent across all four trust measures, with effect sizes ranging from *d* = 0.28 to 0.35 (all *p* < 0.001). Notably, 24.7% of hesitant participants rated themselves as “very trusting” in healthcare professionals, compared to 18.2% of non-hesitant participants (*χ*^2^ = 23.45, *p* < 0.001). Detailed comparisons are presented in Supplementary Table S2. This counterintuitive finding is further interpreted in the Discussion (Section 4.1).

## 4 Discussion

### 4.1 Trust as core driver: beyond information to relationship

Our findings underscore trust, particularly in healthcare professionals, as the strongest predictor of HPV vaccination intention among FCSs, with a clear dose–response gradient. This aligns with the 3C model’s emphasis on “confidence” and extends prior HBM-based work by isolating trust’s unique contribution (17). The strong intercorrelations among trust in healthcare professionals, imported vaccines, and domestic vaccines suggest a holistic “vaccine trust” construct, consistent with trust transfer theory (18): credibility attributed to authoritative sources (medical professionals) generalizes to the products they endorse, reducing perceived risk (19). The clinical relevance of trust extends beyond intention to actual uptake: vaccinated participants in our sample reported substantially higher trust across all measures (Cohen’s *d* = 0.45–0.58) and much lower self-payment hesitancy (18.3% vs. 88.1%) compared to unvaccinated peers (Supplementary Table S1).

A counterintuitive finding also emerged: we observed that hesitant participants reported slightly higher trust scores than non-hesitant participants (Supplementary Table S2). We propose an “information overload with overcaution” hypothesis: these individuals may engage deeply with vaccine information, scrutinizing safety data, following media reports, yet become paralyzed by conflicting details or confirmation bias, focusing on rare adverse events. Their questionnaire responses may reflect cognitive recognition of vaccine benefits (“I know it’s safe”), while emotional or intuitive processing fuels hesitancy (20). This suggests that effective health communication must not only provide information but also help audiences integrate it, avoiding decision paralysis (21). Notably, many participants expressed confusion in open-ended comments despite trusting their doctors, highlighting that trust alone may be insufficient in the face of information overload (22).

### 4.2 Trust buffers price: a prospect theory interpretation

Price ranked as the most frequently cited barrier (53.5%), yet our interaction analysis revealed that high trust nullifies price concerns. This buffering effect, first demonstrated here for HPV vaccine hesitancy, aligns with prospect theory (20): under uncertainty, individuals weigh potential losses more heavily than gains. For those with low trust, vaccine cost represents a certain immediate loss, while future health benefits remain uncertain, making price paramount. High trust reframes the decision: non-vaccination becomes a potential loss (future cancer risk), while vaccination offers certain gain (reduced worry, protection) (23). Trust thus shifts focus from present cost to future benefit.

This insight carries practical weight: in resource-constrained settings where blanket price reductions are infeasible, investments in trust-building (provider communication, transparency about safety data) may yield disproportionate returns by attenuating price sensitivity. Trust cultivation operates as a “leverage point,” amplifying the impact of any subsequent financial support. This study finds it encouraging that even modest trust-building efforts could help bridge the equity gap in vaccine access.

### 4.3 Multidimensional barriers and the “medical student paradox”

The top three hesitancy reasons mapped cleanly onto the 3C model: price (convenience), side effect concerns (confidence), and limited knowledge (confidence/complacency). This supports the 3C framework’s validity in this population and implies that interventions must address all three dimensions synergistically.

The finding that medical students showed lower intention than non-medical peers, dubbed the “medical student paradox” merits reflection. Further analysis revealed that medical students showed comparable trust in imported vaccines but significantly lower trust in domestic bivalent vaccine, suggesting skepticism specifically toward domestically manufactured products, possibly due to heightened awareness of past vaccine safety incidents (e.g., 2018 Changsheng scandal) amplified by medical training. Open-ended comments from medical students frequently cited “rare adverse effects reported in journals” and “incomplete long-term safety data” rather than general concerns about injection pain or immediate reactions. Medical training may foster critical thinking that, without balanced risk communication, tilts toward excessive skepticism. Greater awareness of rare adverse effects, unaccompanied by training in evidence synthesis, may amplify perceived risks. This echoes the “knowledge–attitude–practice” gap: knowing more does not automatically translate into favorable attitudes or actions (24). For future healthcare workers, curricula should explicitly address vaccine risk communication and evidence-based decision-making. As educators, we see this as both a challenge and an opportunity: if we can equip medical students with skills to navigate uncertainty, they will become powerful advocates for vaccination in their communities (25).

### 4.4 From data to action: stratified intervention strategies

The decision tree’s identification of five distinct high-risk (Rule 1–5) profiles enables precision health promotion. Rather than one-size-fits-all messaging, interventions can be tailored to each subgroup’s specific barriers, guided by the 3C dimensions. We can start with rule 1 (highest priority) for a pilot, and then scale it to the other four high-risk groups after evaluation, to ensure efficient use of limited resources. Partnerships with university clinics, student affairs, and local CDC offices can sustain these efforts. We hope that by treating students not as a homogeneous mass but as individuals with unique concerns, we can foster a culture of trust that extends beyond HPV vaccination to other health behaviors.

Rule 1 (Distrust + High price): This core high-risk group (32.4% of hesitant individuals) requires intensive, two-pronged intervention. Build trust through one-on-one consultations with senior clinicians (gynecology, infectious disease) who can address safety concerns authoritatively. Address cost through targeted subsidies or payment plans (26). Trust-building should precede financial discussion, leveraging the buffering effect we documented (27).

Rule 2 (Distrust + Not high price+ Junior): For younger students whose hesitancy stems primarily from distrust, early intervention in orientation programs, “Trust First” modules delivered by trusted healthcare professionals, can prevent skepticism from hardening. Peer-led question and answer sessions may also resonate (28).

Rule 3 (Neutral trust + Low price + Non-medical): This group combines cost concerns with potential information gaps. Peer education by medical students (using accessible language, infographics) can address knowledge barriers, while scholarships or subsidies ease financial strain (29).

Rule 4 (High trust +Low income): Trust is already established; the bottleneck is affordability. Streamlined access to subsidies, insurance education, and clear communication about reimbursement procedures can convert willingness into action.

Rule 5 (High trust + Parent support + Under the age of 22): Young students reliant on parents may need family-level engagement. Personalized risk calculators (showing individualized cervical cancer risk based on age, family history) can increase perceived susceptibility, countering complacency (30). Inviting parents to educational sessions extends trust to the family unit (31).

For the remaining participants (Low-risk group): Ongoing science communication, digital literacy campaigns, streamlined appointment systems, and rapid rumor debunking can create a supportive environment for all students (32, 33). These efforts address the “information overload” challenge by curating trusted, synthesized content.

Beyond subgroup-specific strategies, our findings support a dual-principle approach for university health services: invest in trust-building as a foundational lever that reduces price sensitivity, and provide targeted financial support for low-trust subgroups. Additionally, catchup campaigns should prioritize students aged ≥26 years, who showed markedly higher hesitancy (52–56%).

### 4.5 Theoretical contributions

This study advances theory in three ways: It validates the 3C model’s applicability to HPV vaccine hesitancy among FCSs, refining “confidence” into multi-source trust (healthcare professionals, domestic/imported vaccines) and revealing trust’s “spillover” across sources. The study also updates the contextual framework by referencing the WHO Immunization Agenda 2031 and the 2025 progress report on the Global Strategy for Cervical Cancer Elimination, aligning findings with contemporary global priorities. Additionally, this study demonstrates trust’s buffering effect on price barriers, offering a prospect theory–based explanation that enriches behavioral decision frameworks (27, 34). By shifting from variable-centered to person-centered analysis via decision tree modeling, it identifies actionable subgroups, demonstrating methodological innovation for precision intervention design.

### 4.6 Limitations and future directions

Several limitations warrant acknowledgment. The cross-sectional design precludes causal inference. Unmeasured confounders, such as sexual history, parental education and attitudes, or social media exposure may influence both trust and intention. Future longitudinal or experimental studies are needed to establish causality. Self-report measures may be subject to social desirability bias, potentially overestimating intention. Linkage with actual vaccination records would strengthen validity. Some multi-category variables were collapsed due to low cell counts, possibly losing nuance. The convenience sampling, while yielding a large sample from 12 provinces, primarily covered universities in provincial capitals or developed prefectures, with underrepresentation of Western provinces and remote rural areas. Therefore, our findings are most directly applicable to FCSs in relatively well-resourced university settings; generalizability to more resource-limited regions should be made with caution. The high vaccination rate (76.8%) in our sample suggests possible selection bias toward more health-engaged students, potentially underestimating true hesitancy prevalence in the general FCS population.

Future research should employ longitudinal designs or community-based trials to track trust–behavior relationships over time. Qualitative interviews could illuminate the psychological processes behind the “information overload with overcaution” pattern we observed. Digital health interventions, using algorithms to match individuals’ risk profiles with tailored educational content, hold promise and merit rigorous evaluation. Incorporating more comprehensive measures—such as social media vaccine information exposure scales and parental vaccine attitude assessments—could yield fuller predictive models. Given the analogous roles of trust, perceived barriers, and health communication across preventive behaviors, the 3C model and decision-tree approach developed here may also be transferable to screening for other female conditions. In China, cervical and breast cancer screening are jointly promoted under the national “Free Two-Cancer Screening – Double Ribbon Action” (免费两癌筛查-双丝带行动) program, which faces comparable challenges in public awareness, healthcare provider trust, and service accessibility. Extending our analytical framework to this broader screening context could help identify high-risk subgroups and inform stratified interventions to improve uptake. Despite these limitations, we believe our findings offer a compelling, human-centered perspective on vaccine decision-making. The FCSs who participated in this study shared their hopes, fears, and uncertainties with us; these findings underscore the importance of translating participant insights into targeted interventions.

## 5 Conclusion

This study confirms the 3C model’s utility in understanding HPV vaccine hesitancy among FCSs and provides the first evidence that trust can buffer price-related barriers. Trust, especially in healthcare professionals, emerges as the dominant driver of intention, exhibiting a dose–response relationship. Decision tree analysis identified several high-risk profiles, with “Distrust + High price” being the most critical. These findings support a shift from generic messaging to stratified, precision interventions that address the specific barrier configurations of each subgroup. By integrating trust-building with targeted financial support and broad health education, such approaches hold promise for reducing hesitancy and advancing progress toward cervical cancer elimination goals.

## Data Availability

The original contributions presented in the study are included in the article/Supplementary material, further inquiries can be directed to the corresponding authors.

## References

[1] Singh D Vignat J Lorenzoni V Eslahi M Ginsburg O Lauby-Secretan B . Global estimates of incidence and mortality of cervical cancer in 2020: a baseline analysis of the WHO global cervical Cancer elimination initiative. Lancet Glob Health. (2023) 11:e197–206. doi: 10.1016/S2214-109X(22)00501-0, 36528031 PMC9848409

[2] Ren W Guo X Liu Z Wu Y Peng R Liu H . Burden of female-specific cancers in China from 1990 to 2021: a systematic analysis for the global burden of disease study 2021. Cancer. (2025) 131:e35712. doi: 10.1002/cncr.35712, 39777671 PMC11705200

[3] Moosa-Battey R Masuku P Mayimele N. Human papillomavirus (HPV) as the main cause of cervical and other related cancers: a review. SA Pharm J. (2025) 92:58–62. doi: 10.36303/sapj.1164

[4] Chidebe RC Osayi A Torode JS. The Global Fund, cervical cancer, and HPV infections: what can low-and middle-income countries do to accelerate progress by 2030? EClinicalMedicine. (2030) 81:1–8. doi: 10.1016/j.eclinm.2025.103127, 40083441 PMC11905856

[5] Dempsey RC Wood AM. Perceived social norms and vaccine hesitancy. Curr Dir Psychol Sci. (2025) 34:357–64. doi: 10.1177/09637214251340023

[6] Lee M Gerend MA Whittington KD Collins SK McKinney SL Franca MC . Factors associated with HPV-associated sexual risk behaviors among sexually active college students. J Behav Med. (2024) 47:334–41. doi: 10.1007/s10865-023-00463-1, 38180576

[7] Huang Y Chen C Wang L Wu H Chen T Zhang L. HPV vaccine hesitancy and influencing factors among university students in China: a cross-sectional survey based on the 3Cs model. Int J Environ Res Public Health. (2022) 19:14025. doi: 10.3390/ijerph192114025, 36360905 PMC9657119

[8] Alizadeh L Keshavarz Z Shalbaf A Iranpour S. Iranian women’s perceptions of human papillomavirus and barriers to vaccination: a qualitative study based on the health belief model. BMC Public Health. (2025) 26:1–11. doi: 10.1186/s12889-025-25583-y, 41286815 PMC12764155

[9] Chen C Chen T Huang M Huang Y Zhang L Li P. Factors associated with HPV vaccine hesitancy among college students: a cross-sectional survey based on 3Cs and structural equation model in China. Hum Vaccin Immunother. (2024) 20:2309731. doi: 10.1080/21645515.2024.2309731, 38314749 PMC10854271

[10] Marshall S Fleming A Sahm LJ Moore AC. Identifying intervention strategies to improve HPV vaccine decision-making using behaviour change theory. Vaccine. (2023) 41:1368–77. doi: 10.1016/j.vaccine.2023.01.025, 36669967

[11] Dong B Xu H Qi Y Li Y. Understanding vaccine hesitancy through the lens of trust and the 3C model: evidence from Chinese general social survey 2021. Front Public Health. (2025) 13:1671457. doi: 10.3389/fpubh.2025.1671457, 41103463 PMC12521134

[12] Ma J Lu W Sun S Zhan Y Zhang J Zhang H. The influencing factors in intention making-decision of human papillomavirus vaccine in Chinese college students: a qualitative study. Patient Educ Couns. (2025) 131:108594. doi: 10.1016/j.pec.2024.108594, 39631196

[13] Salleh NS Abdullah KL Chow HY. Cultural barriers and facilitators of the parents for human papillomavirus (HPV) vaccination uptake by their daughters: a systematic review. J Pediatr. (2025) 101:133–49. doi: 10.1016/j.jped.2024.07.012, 39510130 PMC11889688

[14] Lu Y Tang C Xu S Hussain I Zhao W Dong Y . Understanding parental HPV vaccination decision in China through the lens of vaccine hesitancy and preference heterogeneity: a discrete choice experiment. Vaccine. (2026) 76:128307. doi: 10.1016/j.vaccine.2026.128307, 41666789

[15] Zhuang Y Kwang Cheol K Botabara-Yap MJ Zhao K Ramos RIA Cao W. Factors associated with reproductive health and health education participation among female college students in China. Front Public Health. (2025) 13:1627669. doi: 10.3389/fpubh.2025.1627669, 40959616 PMC12433993

[16] Jiang N. Zhang K. Wu M. Song X. Sun W. Wang M. . Exploring the use of the 3C model in the context of HPV vaccine hesitancy in women of childbearing age. Infect Dis Res Учредители: TMR Publishing Group. (2025) 6:3. doi: 10.53388/IDR2025003.

[17] Roy M. From theory to practice: mapping the implementation of the health belief model (HBM) in health-seeking behavior research among black populations in the US. Rev Commun. (2026) 26:96–110. doi: 10.1080/15358593.2026.2616605

[18] Martinelli M Veltri GA. Shared understandings of vaccine hesitancy: how perceived risk and trust in vaccination frame individuals’ vaccine acceptance. PLoS One. (2022) 17:e0276519. doi: 10.1371/journal.pone.0276519, 36269739 PMC9586382

[19] Chen X Wang L Huang Y Zhang L. Risk perception and trust in the relationship between knowledge and HPV vaccine hesitancy among female university students in China: a cross-sectional study. BMC Public Health. (2024) 24:667. doi: 10.1186/s12889-024-18166-w, 38429644 PMC10908003

[20] Burton-Jeangros C. "Anxiety, fear, and panic: the role of emotions in prevention". In: Experiences of Health Risks: Prevention, Power Dynamics and Inequalities. Berlin, Germany: Springer (2025). p. 53–92.

[21] Bauder L Giangobbe K Asgary R. Barriers and gaps in effective health communication at both public health and healthcare delivery levels during epidemics and pandemics; systematic review. Disaster Med Public Health Prep. (2023) 17:e395. doi: 10.1017/dmp.2023.61, 37202845

[22] Shukla AD Goh JM Agarwal L. The illusion of trust and the paradox of disclosure: how fake physician reviews exploit privacy concerns. Internet Res. (2025). doi: 10.1108/intr-01-2024-0042

[23] Andigema AS Olive DPA Vaccine hesitancy as a systems-level vulnerability: historical evolution, sociopolitical drivers, and implications for immunization resilience (2026). doi: 10.20944/preprints202603.0505.v1

[24] Anni NS Rehman N Nyambi A Musiwa A Graham T Dine RD . Knowledge, attitudes, and practices towards human papilloma virus and uptake of HPV vaccine: a protocol for a systematic review. PLoS One. (2024) 19:e0313887. doi: 10.1371/journal.pone.0313887, 39591449 PMC11594429

[25] Murciano-Gamborino C Pérez-Breva L Diez-Domingo J Fons-Martinez J. Promoting HPV vaccination in culturally diverse populations: a reflexive tool for healthcare professionals. Open Res Eur. (2025) 5:381. doi: 10.12688/openreseurope.21810.1

[26] You T Zhao X Pan C Gao M Hu S Liu Y . Informing HPV vaccine pricing for government-funded vaccination in mainland China: a modelling study. Lancet Reg Health West Pac. (2024) 52:1–24. doi: 10.1016/j.lanwpc.2024.101209, 39430124 PMC11489076

[27] Zimmerman AL Modelling the impacts of trust, behaviour, and social determinants of health on HPV vaccination: a simulation study (2025). Available online at: https://hdl.handle.net/10388/17200

[28] Lindsay AC Antunes CV Pires AG Pereira M Nogueira DL. Social ecological influences on HPV vaccination among cape Verdean immigrants in the US: a qualitative study. Vaccine. (2025) 13:713. doi: 10.3390/vaccines13070713, 40733690 PMC12300976

[29] Malik S Mock KO Martillotti R Caravella G Zhou X Mbamelu M . HPV vaccines among university students: understanding barriers and facilitators of vaccine uptake. Vaccine. (2024) 12:1385. doi: 10.3390/vaccines12121385, 39772047 PMC11680171

[30] Curzons S The emotional impact of testing positive for high-risk HPV: an exploration of Cis-women’s experiences following a positive test result in the UK (2025). Available online at: https://uhra.herts.ac.uk/id/eprint/16743/

[31] Çevik HS Amariutei A Mazur A Peker GC Görpelioğlu S Vinker S . Unlocking the key to HPV prevention: exploring factors influencing HPV vaccination decisions among young people and their parents. Public Health. (2025) 238:214–20. doi: 10.1016/j.puhe.2024.12.016, 39689649

[32] Anderson A A scientific literature review: public health messaging and social marketing of the HPV vaccine (2025) 1–9. Available online at: https://scholar.google.com/scholar?hl=en&as_sdt=0%2C5&q=A+scientific+literature+review%3A+public+health+messaging+and+social+market%02ing+of+the+HPV+vaccine&btnG=

[33] Sood RA Carpo BG Leheste JR Carpo B. Bridging the gap: enhancing HPV (human papillomavirus) education to combat rising cancer rates. Cureus. (2024) 16. doi: 10.7759/cureus.74023, 39703290 PMC11658710

[34] Olusanya OA Tomar A Thomas J Alonge K Wigfall LT. Application of the theoretical domains framework to identify factors influencing catch-up HPV vaccinations among male college students in the United States: a review of evidence and recommendations. Vaccine. (2023) 41:3564–76. doi: 10.1016/j.vaccine.2023.04.071, 37164820

