# Supplementary material for: Trust buffers price-related barriers to HPV vaccination among female college students: a cross-sectional study in China based on the 3C model
Source: Front Public Health. 2026 Jul 14;14:1848085. doi: 10.3389/fpubh.2026.1848085 (PMC13407533; doi:10.3389/fpubh.2026.1848085)
Supplement: Supplementary file 1 [file Data_Sheet_1.doc]

# Supplementary Information (SI)

# Trust Buffers Price-Related Barriers to HPV Vaccination Among Female College Students: A Cross-Sectional Study in China Based on the 3C Model

**This file includes:**

1. **Supplementary tables**

**Table 1 Sample Characteristics (N = 4326)**

| **Variable** | **Category** | **n** | **%** |
| --- | --- | --- | --- |
| Age (years), Mean±SD |  |  | 24.64±3.28 |
| Grade | Associate degree | 1182 | 27.3 |
|  | Undergraduate | 2004 | 46.3 |
|  | Master's or higher (postgraduates) | 1140 | 26.4 |
| Household registration | Rural | 2280 | 52.7 |
|  | Urban | 2046 | 47.3 |
| Only child | Yes | 2597 | 60.0 |
|  | No | 1729 | 40.0 |
| Major | Medical | 1856 | 42.9 |
|  | Non-medical | 2470 | 57.1 |
| Income source | Parental support | 2696 | 62.3 |
|  | Part-time income | 737 | 17.0 |
|  | Family/friend support | 304 | 7.0 |
|  | Student loan | 289 | 6.7 |
|  | Scholarship | 199 | 4.6 |
|  | Other | 101 | 2.3 |
| Received HPV vaccine | Yes | 3320 | 76.8 |
|  | No | 1006 | 23.2 |
| Vaccination intention | Very unwilling | 430 | 9.9 |
|  | Somewhat unwilling | 982 | 22.7 |
|  | Uncertain | 869 | 20.1 |
|  | Somewhat willing | 904 | 20.9 |
|  | Very willing | 1141 | 26.4 |
| Self-payment hesitancy | Hesitant | 1494 | 34.5 |
|  | Not hesitant | 2832 | 65.5 |

Note: Age range: 16 to 41 years; percentages are valid percentages.

**Table 2 Significant Univariate Associations with Vaccination Intention**

| **Domain** | **Variable** | **χ²** | **df** | ***P*** | **Cramer's V** |
| --- | --- | --- | --- | --- | --- |
| Demographics | Grade | 149.64 | 8 | <0.001 | 0.13 |
|  | Only child | 11.66 | 4 | 0.020 | 0.05 |
|  | Major background | 9.99 | 4 | 0.041 | 0.05 |
|  | Income source | 115.80 | 40 | <0.001 | 0.11 |
| Trust factors | Trust in domestic bivalent vaccine | 1752.21 | 16 | <0.001 | 0.35 |
|  | Trust in imported HPV vaccine | 1824.16 | 16 | <0.001 | 0.36 |
|  | Trust in healthcare professionals | 1979.04 | 16 | <0.001 | 0.36 |
|  | Trust in preventive efficacy | 1582.95 | 16 | <0.001 | 0.34 |
| Price/behavior | 9-valent price perception | 68.42 | 8 | <0.001 | 0.10 |
|  | 2-valent price perception | 34.42 | 8 | <0.001 | 0.08 |
|  | Acceptable price | 66.35 | 20 | <0.001 | 0.09 |
|  | Self-payment hesitancy | 51.68 | 4 | <0.001 | 0.08 |

### *Note: Cramer's V effect size (0.1 small, 0.3 moderate, 0.5 large). Non-significant factors included household registration (P=0.075) and the necessity of school curriculum (P=0.382).*

**Table 3 Multiple Linear Regression: Vaccination Intention**

| **Variable** | **B** | **SE** | **β** | **t** | ***P*** | **VIF** |
| --- | --- | --- | --- | --- | --- | --- |
| Constant | 1.568 | 0.142 | — | 11.052 | <0.001 | — |
| Healthcare trust | 0.273 | 0.014 | 0.284 | 18.954 | <0.001 | 1.886 |
| Imported vaccine trust | 0.250 | 0.015 | 0.247 | 16.796 | <0.001 | 1.816 |
| Domestic bivalent trust | 0.247 | 0.014 | 0.257 | 17.414 | <0.001 | 1.828 |
| Age | –0.021 | 0.005 | –0.052 | –4.355 | <0.001 | 1.201 |
| Postgraduate (vs. lower) | –0.116 | 0.035 | –0.038 | –3.352 | 0.001 | 1.088 |
| Non-medical major | –0.082 | 0.030 | –0.030 | –2.754 | 0.006 | 1.020 |
| Non-parental income | –0.048 | 0.031 | –0.017 | –1.548 | 0.122 | 1.074 |
| Price acceptability | 0.007 | 0.011 | 0.006 | 0.594 | 0.553 | 1.007 |
| Hesitancy (yes) | 0.009 | 0.031 | 0.003 | 0.295 | 0.768 | 1.042 |

### *Note: R²=0.488, adjusted R²=0.487, F=456.289, P<0.001. Variable coding: Grade (Postgraduate=1, associate degree or undergraduate=0), Specialty (non-medical=1, medical=0), Economic status (other=1, parental support=0), Hesitancy (yes=1, no=0).*

**Table 4 Logistic Regression: Self-Payment Hesitancy**

| **Variable** | **B** | **SE** | **Wald** | ***P*** | **OR** | **95% CI** |
| --- | --- | --- | --- | --- | --- | --- |
| Age | –0.102 | 0.010 | 95.17 | <0.001 | 0.903 | 0.885–0.922 |
| Imported vaccine trust | 0.077 | 0.025 | 9.18 | 0.002 | 1.080 | 1.028–1.135 |
| Non-parental income | –0.172 | 0.071 | 5.93 | 0.015 | 0.842 | 0.733–0.967 |
| Constant | 1.426 | 0.259 | 30.28 | <0.001 | 4.161 | — |

*Note: –2LL = 5032.45; Nagelkerke R² = 0.048; model χ² = 153.62, P < 0.001.*

**Table 5 Reasons for HPV Vaccine Hesitancy (Multiple Responses)**

| **Reason** | **Responses (n)** | **Case %** |
| --- | --- | --- |
| Vaccine price too high | 2314 | 53.5 |
| Fear of side effects | 2280 | 52.7 |
| Lack of vaccine knowledge | 2004 | 46.3 |
| Inconvenient appointment procedures | 1917 | 44.3 |
| Vaccine not widely available | 1763 | 40.8 |
| Believe cervical cancer risk is low | 1744 | 40.3 |
| Doubt vaccine's cancer prevention ability | 1661 | 38.4 |
| Lack of family/friend support | 1468 | 33.9 |
| Fear of injection pain | 1373 | 31.7 |
| Other | 889 | 20.6 |
| Total | 17,413 | 402.5* |

*Note: *Total exceeds 100% due to multiple responses; average 4.0 reasons per person.*

**B.Supplementary figures**

*
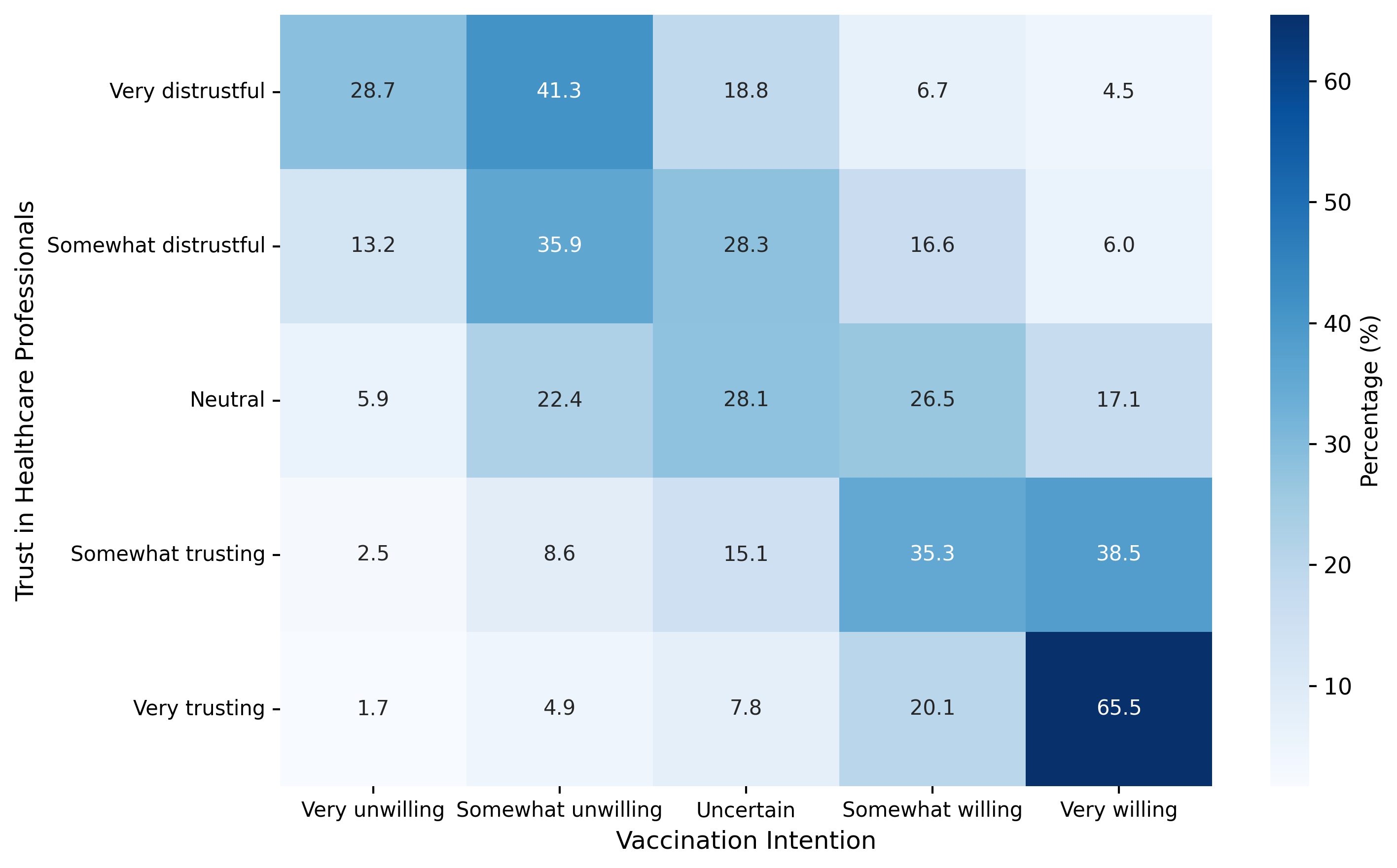
*

*Fig.1. Trust in Healthcare Professionals and Vaccination Intention.*

*χ² = 1979.04, df = 16, P < 0.001; row percentages shown.*


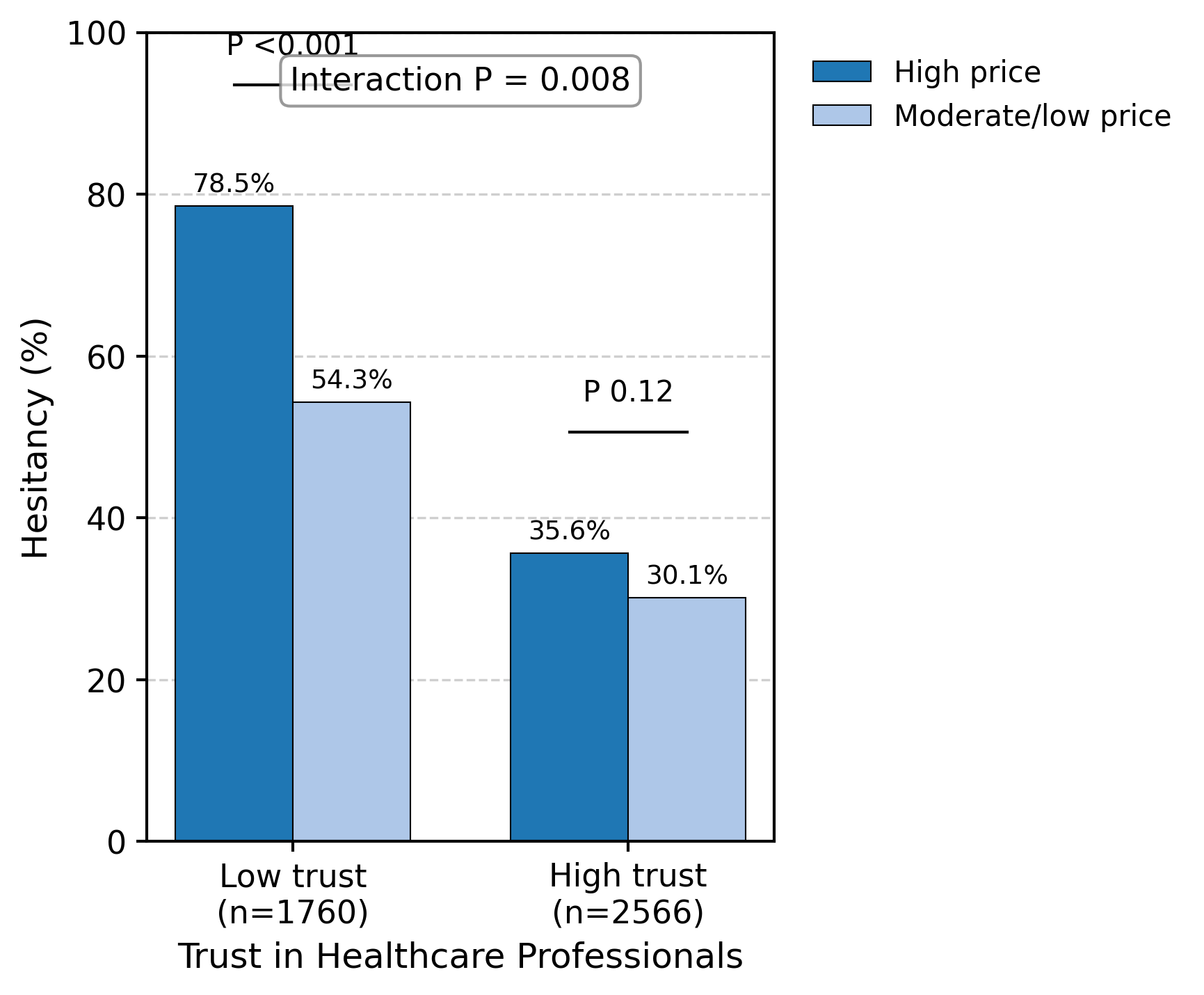


*Fig.2. Interaction Effect between Trust in Healthcare Professionals and Price Perception on HPV Vaccine Hesitancy. Breslow–Day test for interaction: χ² = 7.12, P = 0.008. Low trust group includes respondents who reported being "very distrustful" or "somewhat distrustful"; high trust group includes "somewhat trusting" or "very trusting". High price perception refers to those who rated the vaccine price as "too high"; moderate/low price includes "appropriate" or "too low". Percentages represent the proportion of hesitant individuals within each subgroup.*


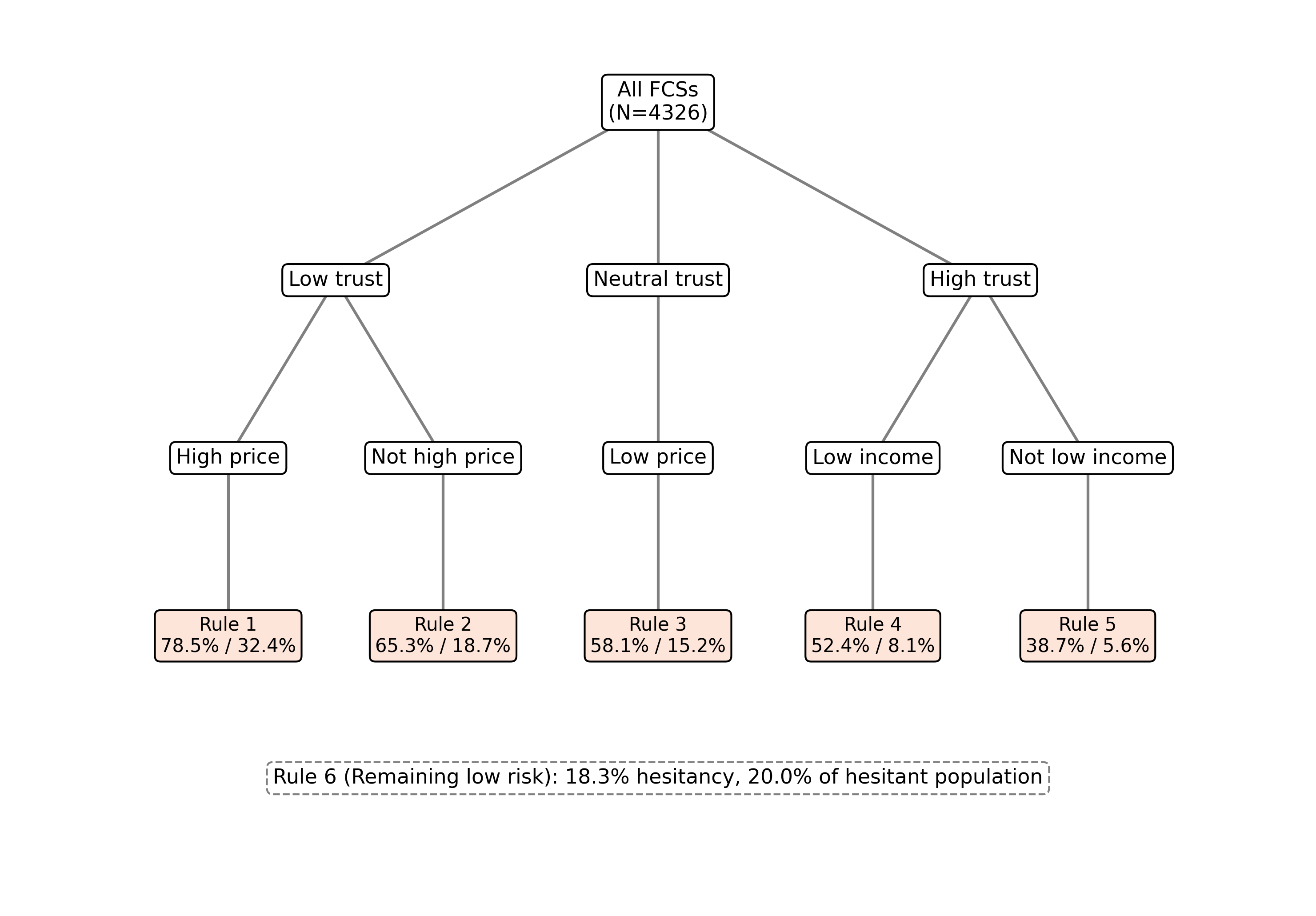


***Figure.3. Classification Rules from Decision Tree Analysis Identifying High-Risk S****ubgroups for HPV Vaccine Hesitancy. CART algorithm; max depth = 5; min parent node = 100; min child node = 50; 10-fold cross-validation. The overall prediction accuracy was 78.3%, with an Area Under the Curve (AUC) of 0.81.*

**
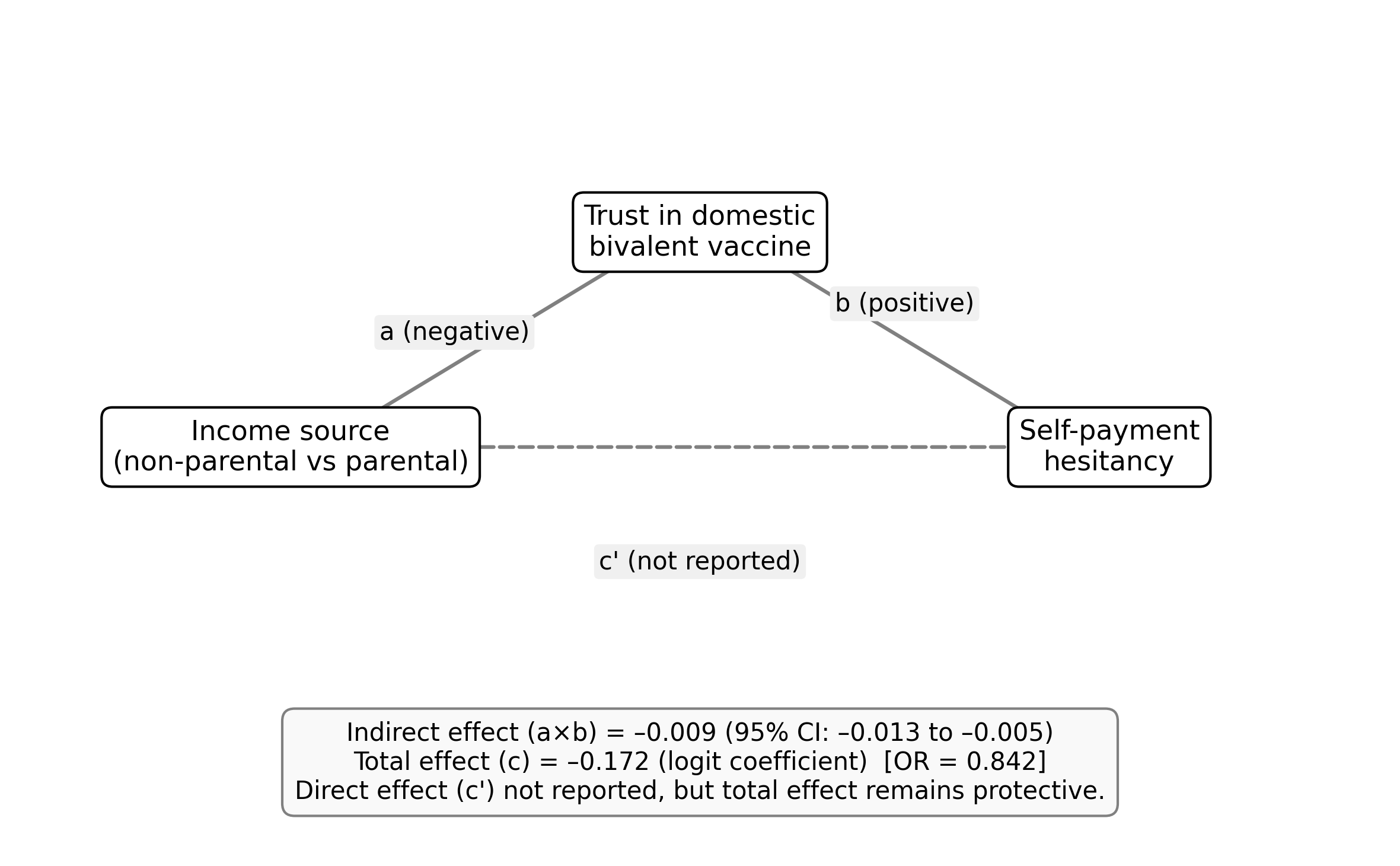
**

*Fig. 4. Mediation pathway of trust in domestic bivalent HPV vaccine between income source and self-payment hesitancy. Standardized path coefficients are not available; unstandardized logit coefficients are shown. The indirect effect (a × b) =–0.009 (95% bootstrap CI: –0.013, –0.005) indicates partial mediation. Total effect (c)=–0.172 (logit coefficient), corresponding to an odds ratio of 0.842 (95% CI: 0.733–0.967). Direct effect (c') was not estimated separately. Solid arrows represent causal paths; dashed arrow denotes direct effect.*
